# Prevalence of Musculoskeletal Disorders in Heavy Vehicle Drivers and Office Workers: A Comparative Analysis Using a Machine Learning Approach

**DOI:** 10.3390/healthcare12242560

**Published:** 2024-12-19

**Authors:** Mohammad Raza, Rajesh Kumar Bhushan, Abid Ali Khan, Abdulelah M. Ali, Abdulrahman Khamaj, Mohammad Mukhtar Alam

**Affiliations:** 1 Department of Mechanical Engineering, National Institute of Technology Manipur, Imphal 795004, India; mohammadrazaamu01@gmail.com (M.R.); rkbnitm@gmail.com (R.K.B.); 2 Department of Mechanical Engineering, Aligarh Muslim University, Aligarh 202001, India; abidak71@yahoo.com; 3 Industrial Engineering Department, College of Engineering and Computer Science, Jazan University, Jazan 45142, Saudi Arabia; abdulrahman@jazanu.edu.sa; 4 Department of Industrial Engineering, College of Engineering, King Khalid University, Abha 61421, Saudi Arabia; mhoda@kku.edu.sa

**Keywords:** lower back pain, knee pain, neck pain, transportation industry, Bayesian network modelling, machine learning

## Abstract

**PURPOSE**: Job profiles such as heavy vehicle drivers and transportation office workers that involve prolonged static and inappropriate postures and forceful exertions often impact an individual’s health, leading to various disorders, most commonly musculoskeletal disorders (MSDs). In the present study, various individual risk factors, such as age, weight, height, BMI, sleep patterns, work experience, smoking status, and alcohol intake, were undertaken to see their influence on MSDs. **METHODS:** The modified version of the Nordic Questionnaire was administered in the present cross-sectional study to collect data from 48 heavy vehicle drivers and 40 transportation office workers. **RESULTS**: The analysis revealed low back pain (LBP), knee pain (KP), and neck pain (NP) to be the dominant pains suffered by the participants from both occupational groups. LBP, KP, and NP were suffered by 56%, 43.75%, and 39% heavy vehicle drivers and 47.5%, 40%, and 27.5% transport office workers, respectively. From the insignificant value of Chi-square, it can be inferred that the participants from both occupations experience similar levels of LBP, KP, and NP. The Bayesian model applied to the total sample showed that NP influenced KP, which further influenced the LBP of the workers. Age was predicted as LBP’s most significant risk factor by the logistic regression model when applied to the total sample, while NP was found to decrease with an increase in per unit sleep. **CONCLUSIONS:** The overall results concluded that heavy vehicle drivers and office workers, irrespective of their different job profiles, endured pain similarly.

## 1. Introduction

In the modern landscape of occupational health and ergonomics, the study of musculoskeletal disorders (MSDs) is a vital concern, affecting the well-being and efficiency of individuals in countless professional domains. MSDs encircle various conditions that affect the muscles, bones, tendons, ligaments, and other parts of the musculoskeletal system [1,2]. These disorders often exhibit discomfort, pain, and functional limitations, significantly influencing the quality of life and job performance of afflicted individuals [2]. Various factors can be attributed to the development of MSDs like age, gender, bad ergonomic posture, poor work conditions, and vibration [3]. If worse, it leads to job absenteeism, increased cost of healthcare, and financial loss suffered by the country [4,5]. In the report released by the European Risk Observatory [6], MSDs were ranked as Europe’s most common occupational medical ailment. In the United States alone, about 32.6% of the population suffers from MSDs, and low back pain (LBP) is the most prevalent [7]. In recent years, India has seen enormous developments in its economy and industrialization, leading to a high prevalence of MSDs among its working class. In Northern India only, it has been reported to be as high as 59.4% [8]. Many physical and psychological ergonomic risk factors exist for most workers, particularly those in the manufacturing, construction, wholesale and retail trade, human health, and social work sectors [9]. Further, a research study revealed that men employed in the construction and manufacturing industries have a higher prevalence of MSDs. In comparison, women employed in hotels and restaurants and the wholesale and retail trade have a higher prevalence of MSDs [10]. Another study was conducted in Canada on over 123 truck drivers, and the researchers reported the prevalence of musculoskeletal pain (MS pain) in 43.1% of drivers over the last year. It was concluded that truck drivers have a higher prevalence of MS pain as compared to, by and large, Quebec male workers [11].

Several epidemiological studies have reported MSDs in different occupational groups such as truck drivers [1,12], taxi drivers [13], bus drivers [14], and dentists [15]. Many studies can be found [12,16] with suggestions for minimizing the MSD pain suffered by heavy vehicle drivers; however, negligible attention has been given to the MSD pain suffered by office workers working in the transportation industry. After an extensive research review, the researcher could find only one study conducted by Mozafari et al. [17], where he compared MSD pain between office workers and truck drivers and found that truck drivers (78.6%) are more prone to MS pain compared to office workers (55.5%). A closely related comparative study was conducted to identify the MSDs in administrative-level staff and bus drivers [14]. Bus drivers were revealed to have significantly more MSDs as compared to their counterparts. The existing literature suggests the prevalence of MSDs among office workers, just like heavy vehicle drivers. The office workers of the transport companies who spend most of their working hours doing their job while sitting complain about MSDs. Next to no study has been conducted on Indian transport office workers in comparison with the MSDs of heavy vehicle drivers. Most studies focus on a single occupational group, either heavy vehicle drivers or office workers, without comparing both [18,19,20]. It is necessary to know if the causes and strengths of MSDs among both groups of workers are similar or different. In India, Niti Aayog published a report mentioning that in 2022, about 4 million trucks, which will rise exponentially to 17 million by 2050, work on roads and highways [21]. This highlights that drivers’ and office workers’ roles and responsibilities have evolved significantly, each playing a crucial role in our modern society, and the grave importance of conducting research using an efficient methodology to relieve them of the causes leading to MSDs.

Following the recent research trends in various scientific disciplines for analyses, the advanced machine learning (ML) approach has been used to uncover complex relationships within datasets. ML is an advanced toolkit that employs algorithms for optimization using training datasets [22]. Deberneh et al. [23], in their research, applied ML techniques for predicting type 2 diabetes. They are also well-suited for finding risk factors for cardiovascular disease [24]. These ML algorithms can reproduce non-linear relations between multiple risk factors well. Hence, they are more suitable for understanding the complex causes of work-related musculoskeletal disorders (WMSDs), ultimately preventing their repetitiveness [25]. One such study was conducted in India by MB Kar et al. [26] on 246 dumper operators to investigate risk factors responsible for WMSDs. They found that random forest (RF) performs better than other algorithms in terms of high accuracy, precision, recall, and F1 score values. Upadhyay et al. [27] conducted a study to investigate the effects of whole-body vibration (WBV) on dumper operators in India. They applied various ML algorithms and concluded that the bootstrap linear regression model was the best-fitted model. The present study focuses on the following occupational groups—heavy vehicle drivers and office workers belonging to the transportation industry. Through the machine learning approach, we aimed to better understand MSDs and associated risk factors with each occupational group, as mentioned above. This novel approach fits perfectly well in the alignment of data analysis development and might provide office workers and heavy vehicle drivers with preventive measures.

For this study, the following hypotheses were framed, which aimed to explore the relationships between MSDs and risk factors (such as age, weight, and occupational habits).

**H_0_1.**  *There would be no significant associations between MSDs and occupational and demographic factors among the total sample of heavy vehicle drivers and office workers.*

**H_0_2.**  *There would be no significant difference in the dominant pains between heavy vehicle drivers and office workers.*

## 2. Methodology

### 2.1. Data Collection Tool

The Nordic questionnaire [28] is a useful tool for epidemiologic studies to determine MSDs. It contains questions regarding the 9 body parts (lower back, neck, shoulders, knees, upper back, hips, elbows, wrists, ankles), asking whether they were painful in the last year. Before collecting the data for the present study, this questionnaire was modified and standardized first and then administered to the sample under study. Keeping in view the understanding level of drivers and workers, the structure of the statements in the NMQ was modified. An additional Body Discomfort Scale, as suggested by experts, while establishing face and content validity, which measured pain severity in the participants (ranging from 0 to 10, where 0 reflects no pain and 10 reflects worst possible pain) for the body parts, i.e., lower back, neck, shoulders, and knees, was included in this study. The modification gave a better picture and understanding of the severity of pain and its causes. A similar modification was also adopted by previous researchers [29,30]. During discussion over the modification of the tool, the experts strongly recommended keeping the focus on these four pain areas, majorly based on their prior research experience in the field; this was also established by Joseph et al. [31] in their systematic review, which revealed that these were the dominant areas where MSDs were most prevalent. Also, the socio-demographic and behavioural characteristics of the participants’ age, weight, height, BMI, work experience, sleeping hours, physical activity status, alcohol consumption status, and tobacco and smoking status were accessed and included as risk factors.

### 2.2. Study Approach

Studies performed by cross-sectional design are appropriate for investigating the relationships between various potential risk factors and the frequency of MSDs [1,32]. The authors carried out this research in accordance with the ethical guidelines presented by the World Medical Association (WMA) in the Declaration of Helsinki. The present study employed a comparative cross-sectional design to investigate the occurrence of MSDs in two distinct occupational groups: heavy vehicle drivers and office workers employed at transportation companies.

Among heavy vehicle drivers, the drivers of four different types of heavy vehicles, i.e., bulldozers, cranes, planners, and trucks, were selected for this study, whereas workers with three different work profiles, i.e., fleet managers, dispatchers, and logistic coordinators working in the office, were selected for this study. The heavy vehicle driver sample selected for this study included the ones who drove a vehicle for long hours at stretches for maximum days in a month. Further, those office workers included in this study who occasionally, as part of their job requirement, drove a heavy vehicle once or twice weekly for small distances, either to park from the main road to the garage or to transport the vehicle from one centre to another nearby centre, which took one to two hours, were included in the control group. Here, the frequency of operating heavy vehicles was controlled. Both the study groups encountered similar corporate policies, work cultures, and industry-specific issues as they were in a similar work environment. This survey employed a random sampling technique for data collection and was conducted along the highways of selected districts of Uttar Pradesh. The topography and climatic features are similar to the rest of the Northern Central Plains of India. The web of highways passing through the study area connects it with all the states of India. The results of this study can be generalized to the drivers of other areas as the working conditions they face are similar to the working conditions faced by the selected sample of heavy vehicle drivers both on the highways and construction sites. The major well-established transport companies located in the selected study areas were chosen for the sample of office workers. Based on the set inclusion criteria by the researchers, the office workers for this study were selected through random sampling. Participants who had at least 5 years of work experience [33] and could communicate in English or Hindi were included. The exclusion criteria comprised the absence of any medical history of surgery on any body part, lack of MSDs prior to commencing driving, and not suffering from any injury due to an accident. A total of 144 eligible participants, out of whom 77 were heavy vehicle drivers of various vehicles running on Indian roads and 65 were office workers from transport companies, were successfully approached and asked to participate in this study. A final sample of 88 participants, of which 48 were heavy vehicle drivers (i.e., 6 bulldozers, 5 cranes, 7 planners’ operators, and 30 truck drivers) and 40 transportation office workers (i.e., 15 fleet managers, 10 dispatchers, and 15 logistic coordinators), corresponding to a response rate of 62.3% and 62.5%, respectively, were selected for this study. Proper consent was obtained from each volunteer who agreed to participate willingly before every start.

### 2.3. Standardization of Tool

Ten experts, including scientific researchers and professors who had vast experience conducting research in the field of design and ergonomics, were selected to establish the face validity and content validity of the modified NQ. They rated the modified NQ on a Likert scale from 1 (strongly disagree) to 4 (strongly agree) to establish face validity, providing their assessment on whether the questionnaire correctly measures personnel’s medical condition. An average score of 3.5 out of 4 was obtained after most of the experts passed the instrument. Changes were made according to the feedback to enhance the clarity of the questionnaire. The suggestions from the experts were related to the language of the statements based on the understanding of the drivers, ambiguousness of the statements, grammatical errors, and restructuring of some statements. These discrepancies were addressed by the researchers themselves, and the questionnaire was again given to the experts for the final validation. After that, the questionnaire also underwent content validation, where lucidity and relevancy were the attributes associated with it. The experts reviewed each item, providing their judgment on a 4-point Likert scale (1 = not relevant, 2 = somewhat relevant, 3 = quite relevant, and 4 = highly relevant). The Item Content Validity Index (I-CVI) was computed for each questionnaire item, whereas the Scale Content Validity Index (S-CVI/Avg.) and Universal Agreement (UA) were calculated overall for both attributes. All values of S-CVI/Avg. and UA were acceptable values observed to be above 0.8. While calculating S-CVI/Avg., Likert scale values 1 and 2 were combined to 0, signifying items considered undesirable, and 3 and 4 were combined to 1, signifying items as desirable. Reliability was assessed by Cronbach’s alpha method implemented with the Pingouin library in Python on the data collected after the pilot study on 25 heavy vehicle drivers and 20 office workers. The resulting values for both were above 0.75, establishing a high internal consistency of the tool. All these systematic assessments finally resulted in a standardized tool ensuring the delivery of consistent and accurate measurements.

### 2.4. Data Exploration by Machine Learning

To investigate the prevalence of MSDs among heavy vehicle drivers and office workers, we explored the data using machine learning algorithms in Python 3.7.0. Our approach encompassed preprocessing the collected data (using the standardized tool), including data cleaning, handling missing values, encoding categorical variables, and scaling numerical features, ensuring the dataset’s quality and consistency during machine learning analysis. Descriptive statistics were utilized for continuous variables, and the results were presented as mean, median, and standard deviation, whereas for categorical variables, the findings were presented as frequency and percentage, as shown in Table 1 and Table 2, respectively. While collecting the data during the conversations with heavy vehicle drivers and office workers, both of the groups complained that LBP, KP, and NP were at times so severe that they caused a hindrance in discharging their duties, while shoulder pain and the other pains did not limit them from doing their work. Hence, only those pains that emerged as being agonizing to the participants dominantly were considered for further statistical analysis. The Chi-square test was conducted utilising the ‘scipy.stats’ library to assess the association between MSDs (LBP, KP, and NP) and the two occupational groups (Table 3). A logistic regression algorithm was applied between universal risk factors, such as age, weight, height, sleep, smoking status, alcohol consumption status, and work experience, and occupational risk factors, whether being a regular heavy vehicle driver or not (coded as 1 and 0, respectively), and the occurrence of MSDs in the combined group of heavy vehicle drivers and office workers to examine the relationships within the last 12 months. The main reason for combining both groups is to see whether being a regular driver is significantly associated with MSDs. The one-hot encoding method was used to represent categorical variables as binary vectors. For our relatively modest dataset size of 88 observations, logistic regression is the best-suited method as it is less likely to overfit the training data than other complex algorithms, and it also has a straightforward interpretation of coefficients and odds ratios. Complex models like random forests, decision trees, or neural networks are at a greater risk of overfitting. These models can learn the noise in the data rather than the underlying patterns, leading to poor generalization to new data. Models were fitted for LBP, KP, and NP, as these were the dominant pains in both groups during our analysis. Eventually, we divided our dataset into 70% training (62 datasets) and 30% testing (26 datasets) sets to evaluate the performance of our ML models. The analysis used the ‘pandas’ library to manipulate data and the ‘scikit-learn’ library for ML tasks. Table 4 shows the adjusted odds ratio (AOR) for each independent variable, providing their effects on the likelihood of the outcome. AOR was calculated from the coefficients obtained from the model fitted on the training dataset. This approach avoids overfitting and ensures that results generalize well to new, unseen data. Multicollinearity was checked among the continuous risk factors. The pseudo R-squared, log-likelihood of the model, log-likelihood Null value, and likelihood ratio test (*p*-value) are reported and shown in Table 4. The significance of findings was tested at a significance level of 5% (α = 0.05) for all the statistical tests.

### 2.5. Ethical Approval and Informed Consent

This study was conducted according to the guidelines of the Declaration of Helsinki and approved by the Department of Mechanical Engineering Institutional Review Committee, National Institute of Technology, Manipur, India (No. NITM.12/(18-ME)/EC/2024-01). Data were gathered between 1 and 30 August 2022. Written Informed Consent was obtained from all participants before participation.

## 3. Results

The present study investigates the impact of risk factors on the likelihood of occurrence of MSDs in the combined group of heavy vehicle drivers and office workers. Significantly associated variables were identified through AOR and their associated *p*-values. The ML model performance was assessed by evaluation metrics (accuracy, precision, recall value, F-1 score, cost function, and receiver operating characteristic curve (ROC)).

### 3.1. General and Behavioural Characteristics

A significant difference between the mean of the general characteristics such as age, work experience, weight, height, BMI, and sleep duration can be seen in Table 1 among both the sub-samples. The minimum age and maximum age of the office workers and drivers were similar, i.e., 21 and 23 years and 62 and 65 years, respectively. While a minimum of 5 years and a maximum of 40 years of work experience were recorded. The participants in both samples were unbiasedly randomly selected; the results revealed that the mean age and mean work experience of the heavy vehicle drivers were 44.02 years and 18.93 years, respectively, while for the office workers, the mean age was 34.57 years and the mean work experience was 12.15 years. Moreover, the mean sleep duration of the drivers was found (5.68 h) to be significantly less than their counterparts, i.e., the office workers (7.1 h). The comparison in Table 2 between the behavioural characteristics revealed that the office workers were engaged in significantly more regular exercises (32.5%) than the drivers (12.5%). Further, the heavy vehicle drivers (52.1%) divulged to consume more alcoholic beverages than the office workers (15%).

### 3.2. MSDs Distribution

**Figure 1:**
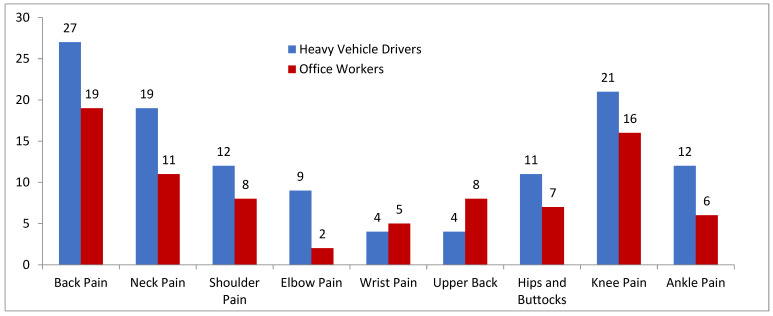
MSD prevalence among the drivers and office workers by body part. (Bar graph showing the MSD among drivers and office workers by body part). Note: Data from Mohammad Raza et al., 2024 [16].

A perusal of Figure 1 shows that 87.5% of the heavy vehicle drivers and 67.5% of the office workers experience at least one type of MSD. In the present study, only LBP, KP, and NP emerged as dominant pains experienced by all the subjects. LBP was reported to be experienced most by both the drivers and workers, with 56.25% and 47.5% of the samples, respectively. The second dominant pain was KP, experienced by 43.75% of the drivers and 40% of the office workers, followed by NP, where 39.58% of the drivers and 27.5% of the office workers complained about it. Among both the samples, i.e., the drivers and office workers, SP (25% and 20%), hip and buttock pain (22.9% and 17.5%), and ankle pain (25% and 15%) were moderately observed, respectively. The pains least experienced by the drivers were wrist pain and upper back pain, both of which were complained about by 8.3% of the drivers, while for the office workers, elbow pain occurred the least in them, marked only by 5%. The Chi-square application in Table 3 showed no statistically significant difference in all three dominant pain types among the samples under study. It can be inferred that the drivers and office workers experienced similar levels of LBP, KP, and NP. From Table 4, it can be inferred that among the various demographic characteristics taken under study, only age came out as a significant risk factor associated with LBP, while sleep duration emerged as the risk factor significantly associated with NP. Also, the *t*-test results in Table 1 revealed no statistically significant difference between the pain severity scores among the samples for LBP, NP, SP, and KP.

### 3.3. Model Summaries

The logistic regression (LR) models were developed to predict the occurrence of LBP, KP, and NP in the combined group of the heavy vehicle drivers and office workers. The grid search methodology was used to conduct comprehensive hyperparameter tuning to optimize the performance of the LBP and KP models. For the NP model, default hyperparameters were used (as shown in Table 5). The evaluation metrics for the train and test datasets are presented in Table 6. To assess the generalization ability of the model, we performed a 10-fold cross-validation on the training set for the LBP and KP models and a 5-fold Stratified Cross-Validation for the NP model. The 5-fold Stratified Cross-Validation technique was used in order to ensure a balance between bias and variance and to account for any class imbalance (Table 6). Test set metrics provide the performance of LR models on unseen data. The prediction model for LBP achieved an accuracy of 0.63, which indicates that the model correctly predicts the target labels for 63% of the test data samples. The precision score of 0.73 reveals that the model is correct about 73% of the time when a positive instance is predicted by the model. The recall score of 0.54, however, suggests that the model only correctly identifies 54% of the positive instances belonging to the target class. The F1 score of 0.62, which is a moderate score, suggests that there is room for improvement. Overall, the prediction model works reasonably well. An accuracy of 0.56 was observed in the LR model predicting KP, which signifies that the model makes correct predictions approximately 56% of the time. However, a precision of 0.47 was reported. This case suggests that out of all predicted positive instances, 47% are actually true cases of KP. On the other hand, a higher value of the recall score of 0.64 and an F1 score of 0.54 were noted. Here, recall, also known as sensitivity, indicates that approximately 64% of the true cases of KP are correctly identifiable by our model. Also, in our case, the F1 score reveals that the model attains overall moderate performance. Furthermore, the prediction model for NP achieved an accuracy of 0.59, and its precision, recall, and F1 score were 0.4, 0.45, and 0.42, respectively, based on the test dataset. The accuracy metric suggests that predictions made by the LR model are approximately 59% accurate. The precision score in our instance reveals that approximately 40% of all the predicted positive instances are actually true cases of NP. Similarly, the low recall value shows that the LR model neglects a significant number of real positive instances, as it is only able to correctly identify 45% of the true cases of KP. Additionally, the F1 score, calculated as the harmonic mean of precision and recall values, tells us that the model achieves moderate performance.

### 3.4. Associations Between Low Back Pain, Knee Pain, and Neck Pain Predicted by Bayesian Network Modelling

**Figure 2:**
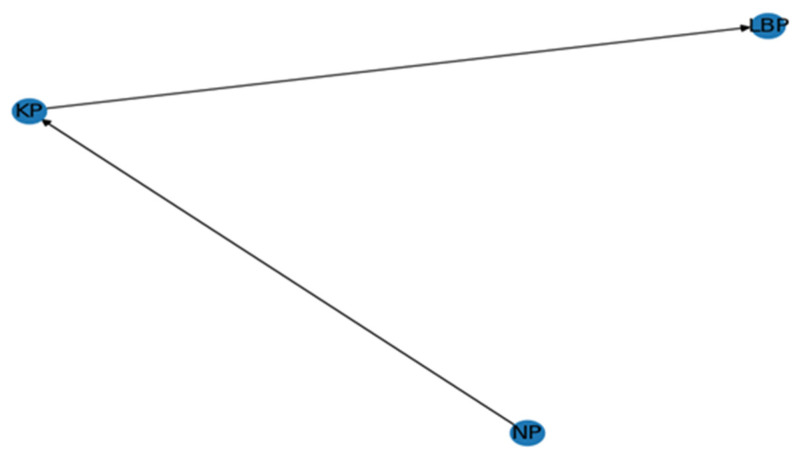
Bayesian network modelling (Bayesian network causal association model between LBP, NP, and KP).

Bayesian network modelling was used to investigate the causal relationships and dependencies between variables—low back pain, knee pain, and neck pain—in the combined group. By using this model, we can see and measure the conditional dependencies between LBP, NP, and KP that can be visualized and measured, further revealing how one condition might affect another over time. Modelling was performed in Python using the ‘pgmpy’ and ‘networkx’ libraries for the construction and interpretation of Bayesian networks. From Figure 2, it can be inferred that the model revealed the causal relationships between the pains, where one pain influenced the body so much that it led to the arousal of another pain. Among the pains taken under study, it was seen in the model that NP was not influenced by the other two pains, but it did have a direct influence on KP. Further, KP has a notable influence on LBP. Seemingly, it can be inferred that KP acts as the mediating pain between NP and LBP. However, the model could not find compelling evidence pointing to a direct causal association between LBP and the other variables.

## 4. Discussion

The present study is a cross-sectional study that was conducted to determine the severity of MSDs experienced by the participants in the last year. It intended to identify the risk factors associated with MSDs among heavy vehicle drivers and transport office workers. The associations found can be used as a foundation for future research, including longitudinal studies that further investigate the causal relationships. During the pilot study, it was observed that the people who had more than 5 years of work experience were the ones who complained the most about pain, and thus, they were approached to participate in this study. A similar trend was also observed by Boshuizen et al. [33]. Despite facing participant refusal, terminations, and flawed responses, where the participants appeared to exaggerate the pain experienced from their MSDs, a final sample of 48 heavy vehicle drivers and 40 office workers was finalized for this study.

In contrast to earlier research that often employed descriptive statistics or conventional regression techniques to investigate MSDs [34], the present work offers machine learning as a fresh analytical approach, improving the understanding of MSD risk variables. More advanced identification of subtle patterns and risk predictions are performed by using machine learning techniques such as logistic regression and other predictive modelling tools. Compared to established traditional statistical tools like SPSS, the machine learning approaches employed in this study offered substantial advantages. The main focus of SPSS was on explanatory models, which aided in the explanation of correlations between variables. Machine learning, however, places a strong emphasis on predictive modelling, as it can accurately predict outcomes (such as who is likely to acquire MSDs) from the given data. Furthermore, its cross-validation features ensure that the results are reliable and can be generalized to a wider range of situations. This increases the findings’ robustness and makes sure that they are not overfit to a certain dataset. There is not as much built-in cross-validation in SPSS.

The results showed that MSD prevalence in both study groups in the last year was high. A total of 87.5% of the heavy vehicle drivers and 67.5% of the office workers complained of at least one type of MSD. Only a few direct studies are available in the literature that compare MSDs in drivers and office workers. One such study, conducted in Iran [17], reported a prevalence rate of MSD of about 55.5% in office workers, which is much below that reported in our study. Tamrin et al.’s [20] study on bus drivers and Robb et al.’s [35] study on truck drivers registered a prevalence rate of total MSDs that was roughly 81.8% and 81%, respectively, comparable to what we projected from our study on heavy vehicle drivers. The variation in the outcomes could be due to environmental factors and demographic characteristics. The most dominant MSDs in both study groups, according to the current results, were LBP, KP, and NP, which were distributed as follows: 56.6%, 43.5%, and 38.1% in heavy vehicle drivers and 47.5%, 40%, and 27.5% in office workers.

The researcher noted that the suspension seats of the drivers were not in ideal condition, and because of this, it was not possible to minimize the adverse effect of the jerks from reaching the driver, which in turn severely affected their lower backs. It was further observed that the high temperature of the driver’s cabin was also beyond the comfortable tolerance range, causing heat stress, adversely affecting health, and causing MSDs. The researcher measured the temperature of the driver’s cabin using a room thermometer, which was more than 38 °C while collecting the data. The observed temperature was a lot higher than the comfortable temperature range of 20–25 °C, as mentioned by Chad et al. [36] in their study. It can be hypothesized that this gives rise to unpleasant sitting postures, leading to repeated changes with frequent braking, further contributing to a twisted knee and stress on the neck. This, in turn, may give rise to pain in both the knee and the neck. Furthermore, the present study revealed that NP emerges as the second most common MSD among drivers. A rigorous systematic review of the literature reported LBP to be the most dominant pain, followed by NP [31]. Because vibration from holding the steering while driving enters the neck through the hands and shoulders, hand–arm vibration may be the cause of NP. The drivers’ adoption of the forward head posture as a result of extended sitting may be another factor of NP [37].

Transport office workers in our study were also occasional drivers of heavy vehicles, which may have impacted an increased rate of MSDs in addition to variations in environmental factors and demographic characteristics. Among them, the probable cause of LBP emerging as the major pain could be due to bad sitting postures that give rise to stiffness and pain [38]. The forward head posture among the workers sitting for longer hours causes neck muscles to harden up, causing NP [39,40]. Long sitting hours in the same postures at times also put pressure on the kneecaps, giving rise to discomfort [41]. When this discomfort extends for longer periods, it ultimately gives rise to knee pain [41]. In previous studies, Arora SN et al. also reported a high prevalence of NP, LBP, and KP among sitting workers [42]. When these workers are subjected to occasional heavy vehicle driving, they are exposed to the jerks and the pressures of both the vehicle and the road. Their bodies are not adapted to safeguard them from sudden jerks. These jerks cast heavy blows and pressure on their joints and vertebral column, giving rise to sudden pain [3]. Workers complained that entering and exiting the cabin without proper ergonomics further strains the knees and lower back. They are subjected to intense heat and cold in the summer and winter, respectively, because their offices are typically outside and lack air conditioning, which exacerbates MSDs [36]. Office workers’ LBP are further impacted by the unloading and loading of heavy vehicles as part of their job description [11,43]. Furthermore, lifting heavy objects by hand puts needless strain on their knees, which exacerbates KP, as predicted in a previous study by Herquelot et al. [44].

The Chi-square test was applied to LBP, NP, and KP between the drivers and workers, and the Chi-square values came out to be 0.364, 0.930, and 0.019, respectively, which were all found to be statistically insignificant (Table 3). This implies that there exists no significant difference in all three pains experienced by both of the groups, i.e., they were experiencing similar levels of pain. Hence, it can be said that the null hypothesis (H_0_2) is accepted.

**Figure 3:**
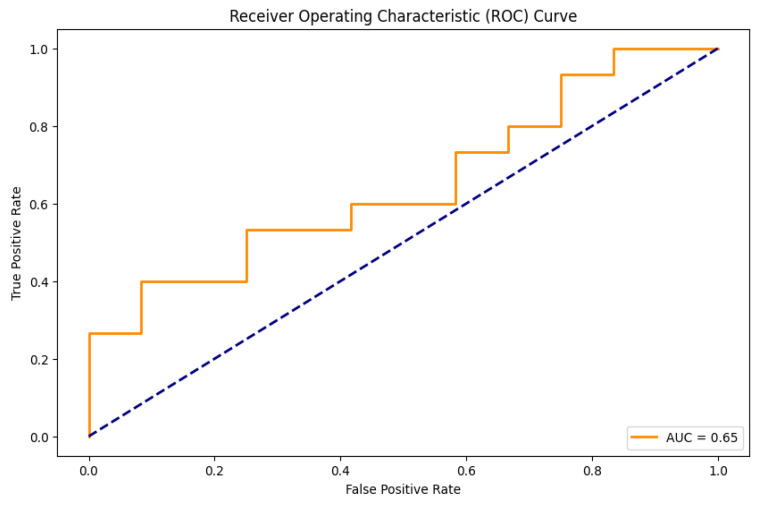
ROC curve on the test set for LBP. (Curve between the true positive rate and the false positive rate).

**Figure 4:**
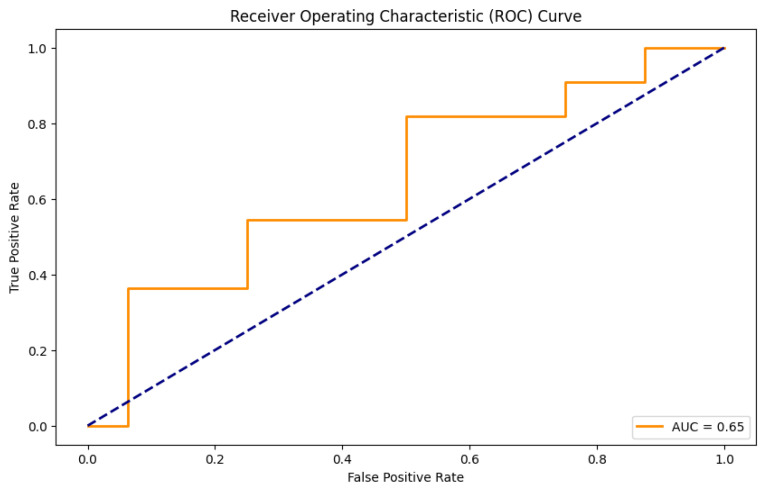
ROC curve on the test set for KP. (Curve between the true positive rate and the false positive rate).

**Figure 5:**
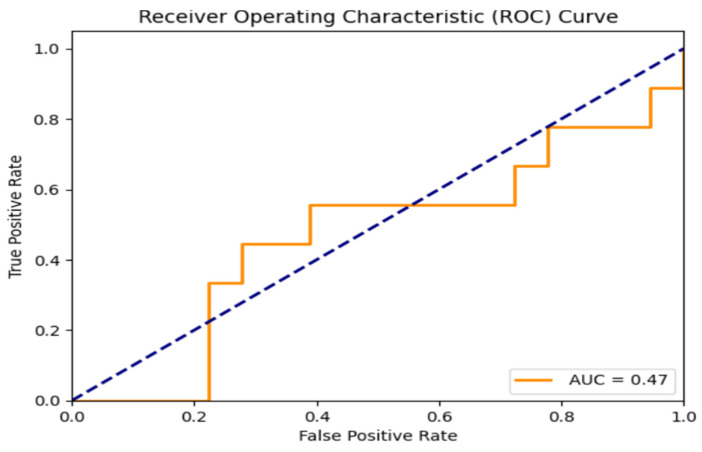
ROC curve on the test set for NP. (Curve between the true positive rate and the false positive rate).

In our study, the performance of LR models built for predicting LBP, KP, and NP was assessed using the different parameters of evaluation metrics, including the area under the receiver operating characteristic curve (AUC). For the NP model, the hyperparameters found after the grid search did not provide the best values for the parameters of the evaluation metrics. Instead, default hyperparameters were used to build the satisfactory model (Table 5). The class weighting parameter was used in the case of the NP model to ensure no bias towards the majority class and effective learning from the minority class. It is performed to balance the importance of both classes. Figure 3, Figure 4 and Figure 5 show ROC curves (AUC) of LBP, KP, and NP, which are used as a measure of model discrimination. AUC values of 0.65 for both LBP and KP reveal that the LR models demonstrated moderate discriminative ability in identifying subjects with or without these MSDs (Table 6). However, for NP, the model provides an AUC of 0.47, which indicates below-average discriminative ability in accurately predicting the subjects who suffered from NP than those who did not. It is noticeable that even with the use of improvement techniques such as the class weighting parameter, which may help improve the prediction of less frequent outcomes, and regularization techniques to prevent overfitting and generalizability, the evaluation metrics still only show moderate performance, especially for neck pain prediction. This may be due to the fact that some risk factors for MSDs, such as posture, ergonomic conditions, or psychosocial factors like stress, were not included in the present data. These factors may have some role in causing neck pain, which is often a result of the combination of lifestyle and work-related influences. Furthermore, linear models like logistic regression are, at times, unable to adequately reflect the complexity of the interactions between risk variables and MSDs.

The findings further revealed that increasing age achieves statistical significance (*p* = 0.008) and is the only significant predictor in the LBP prediction model (Table 4). According to our analysis, the combined group’s chance of having LBP increased by 1.14 times for each unit rise in age in years. This outcome was also suggested by Rehman et al. [45] in their study, which states that age is a significant risk variable for the occurrence of WMSDs. Also, sleep duration (*p* = 0.041) comes out to be the only significant predictor in the NP model. The probability of getting NP decreases by 0.546 times for each unit increase in sleep. Longer sleep hours allow for muscle relaxation and reduce inflammation. A previous study [46] revealed that sleep disturbance increases the probability of NP. Thus, the null hypothesis (H_0_1) was rejected. Furthermore, our analysis of the ML models predicting KP found no statistically significant risk factors associated with them, which was not expected. BMI and work experience are the most well-established risk factors associated with MSDs [47], but our analysis predicts otherwise. Among heavy vehicle drivers, rather than BMI, whole-body vibration exposure may be the main cause of MSDs, which makes vibration exposure a more significant component. Similarly, in the office workers, too, rather than BMI, sedentary posture and behaviour were found to be more important contributors to MSDs. Hence, while examining a combined group with different exposures in their jobs, weight and height (or BMI) might not be as significant a factor as sought. Both groups may have different physical effects from their work experience. While office professionals with greater work experience may have better ergonomic setups or have adapted to their sedentary job conditions, heavy truck drivers may develop MSDs as a result of prolonged exposure to whole-body vibration and harsh vehicle surroundings. This variability in how work experience affects MSDs across job types may explain the lack of significance in the combined analysis. It is important to consider the study demographic, sample size, measuring methods, and potential confounding variables as they may have affected the outcome. These results demonstrate the complexity of MSDs and emphasize the need for more investigation to fully comprehend these disorders’ multifaceted nature.

To determine the potential relationships between LBP, KP, and NP, a Bayesian model was applied, which showed that NP influenced KP, and, in turn, KP influenced LBP. The possible explanation for this could be that sitting for long hours on an uncomfortable seat and in bad posture leads to stiffness of the neck muscles, giving rise to sharp pain in the neck. A little jerk while walking or moving increases the sudden intensity of the pain by many folds. To avoid such piercing pain in the neck, a person usually tries to adopt several altered walking patterns that deviate from normal walking, which affects alignment and the body weight distribution on the legs, causing increased stress on the knees. This increased stress may lead to knee pain. NP leading to osteoarthritis is a very less explored area. The only study found by the researchers was by Chen et al. [48], who found a proportional relationship between NP and ailment. Knee pain further decreases the normal functioning of the subject. The reduced movement and limited physical activities impact the person’s lower back, causing pain. To find a comfortable sitting position, the person tries all possible means, the burden of which is endured by the lower back muscles, which may result in added pain [49,50]. Comprehension of these causal pathways will make it possible to prioritize interventions that address the root causes of pain rather than just the symptoms. In order to create interventions (such as physical therapy, ergonomic changes, or medical treatment) that will not only address individual symptoms but also look into the more general patterns of pain among their patients, clinicians and practitioners will find this knowledge helpful in developing comprehensive management strategies for drivers with musculoskeletal disorders. We could not have known these specific risk chains without machine learning analysis.

### Practical Recommendations

Drivers should reduce their long driving time and take breaks between driving after prolonged exposures. The vehicles’ suspension should be checked and improved routinely to avoid excessive vibration. Seat cushioning should be adjusted by drivers as per their ease, which could help with vibration reduction. Lumbar support and inclination of the backrest should be at the proper degree with the back. If suffering from any pain, then long hours of driving and driving on rough terrain should be avoided. The seating of office workers should be such that their body posture is maintained, and they need not lean forward too much to work. Experts should give them proper training on using safe manual handling techniques, as they occasionally load and unload vehicles, preventing knee and lower back strain. Training programs show promising results in reducing the prevalence of MSDs [51].

The present study suggests future research where larger samples from diverse populations can be taken, and machine learning can be used, leading to results that can be generalized across different industries and occupations.

## 5. Limitations

Due to the cross-sectional nature of the present study, the establishment of causal inferences is restricted. To develop temporal relationships and causality between risk factors and MSDs, more comprehensive longitudinal studies are required. Furthermore, with dependency on self-reported data for MSDs and risk factors, there is a risk of introducing recall bias and social desirability bias. This study used a random sampling technique along highways and transport companies in selected districts of Uttar Pradesh, which may not have captured the larger population of heavy vehicle drivers and office workers. Other confounding factors, such as specific job tasks or ergonomic interventions, were not taken into account in this study, which could influence the prevalence of MSDs in both groups. Also, here, for the analysis parts, we used logistic regression models only. Other sophisticated models and validation techniques are needed besides logistic regression models for more robust predictions. Further, the present study only evaluates the effects of individual risk factors on MSDs in the combined group. Other physical risk factors that may be common to both groups have not been taken into account. Additionally, the use of self-reported symptoms rather than clinical diagnoses could be another limitation in this study. An accurate diagnosis and medical examination are necessary for the accurate description of MSDs [52]; however, simple methods such as self-modified questionnaires are frequently employed to detect these conditions [53]. Thus, to safeguard vulnerable workers, future studies should concentrate on the necessity of prevention and treatment programs.

## 6. Conclusions

The present study aimed to understand MSDs and the risk factors that were associated with heavy vehicle drivers and office workers working in the transportation industry. Among both the job profiles, LBP was found to be the dominant pain, followed by KP then NP. Increasing age and shorter sleep duration were identified as significant risk factors among all the participants in predicting the likelihood of LBP and NP, respectively, thus rejecting the first null hypothesis, H_0_1. The Bayesian network model applied to the total sample revealed the existence of a direct causal association, where KP was observed to be potentially influenced by NP, which was, in turn, found to influence LBP. Further, the Chi-square test revealed that no significant difference was observed between the sample of workers with respect to LBP, KP, and NP, thus accepting the null hypothesis H_0_2. The results suggest that proper ergonomic considerations should be implemented in workers’ workplaces to reduce the long-term effect of MSDs on the body despite their increasing age.

## Figures and Tables

**Table 1:** General characteristics and pain severity.

Characteristics	Heavy Vehicle Drivers		Office Workers		*p*-Value *
	Mean ± SD	Median	Mean ± SD	Median	
Age (years)	44.02 ± 12.91	44	34.57 ± 10.4	33	**0.0004**
Work Experience (years)	18.93 ± 10.61	19	12.15 ± 9.085	8.5	**0.002**
Weight (kg)	65.14 ± 10.90	64.5	69.72 ± 12.28	70	0.067
Height (m)	1.66 ± 0.071	1.66	1.686 ± 0.089	1.65	0.192
Body Mass Index (BMI)	23.56 ± 3.97	23.66	24.54 ± 4.11	24.42	0.262
Sleep Duration (hours)	5.68 ± 2.06	5	7.1 ± 1.23	7	**0.0003**
Low Back Pain Severity *	3.29 ± 3.08	4.5	3.175 ± 3.21	5	0.862
Neck Pain Severity *	2.10 ± 2.67	0	2.125 ± 3.17	0	0.973
Shoulder Pain Severity *	1.33 ± 2.37	0	1.4 ± 2.76	0	0.903
Knee Pain Severity *	2.96 ± 0.64	3	2.93 ± 0.69	3	0.823

* As per two-sample independent *t*-test. * On the pain severity scale, 1 = no pain and 10 = Worst Imaginable Pain. Note: Data from Mohammad Raza et al., 2024 [16].

**Table 2:** Behavioural characteristics.

	Heavy Vehicle Drivers		Office Workers		*p*-Value *
	Frequency (n) (Yes/No)	Percentage (%) (Yes/No)	Frequency (n) (Yes/No)	Percentage (%) (Yes/No)	
Do you exercise regularly?	6 (42)	12.5 (87.5)	13 (27)	32.5 (67.5)	**0.023**
Do you smoke or chew tobacco?	40 (8)	83.33 (16.67)	11 (29)	27.5 (72.5)	0.180
Do you drink alcoholic beverages?	25 (23)	52.1 (47.9)	6 (34)	15 (85)	**0.0001**

* As per the two-sample *Z*-test for proportions. Note: Data from Mohammad Raza et al., 2024 [16].

**Table 3:** Classification of study groups based on the type of pain.

MSD Pain	Heavy Vehicle Drivers		Office Workers		Chi-Square Statistic	*p*-Value *
	Frequency (n) (Yes/No)	Percentage (%) (Yes/No)	Frequency (n) (Yes/No)	Percentage (%) (Yes/No)		
Low Back Pain	27 (21)	56.25 (43.75)	19 (21)	47.5 (52.5)	0.364	0.545
Neck Pain	19 (29)	39.5 (60.5)	11 (29)	27.5 (72.5)	0.930	0.334
Knee Pain	21 (27)	43.75 (56.25)	16 (24)	40 (60)	0.019	0.890

* As per the Chi-square test. Note: Data from Mohammad Raza et al., 2024 [16].

**Table 4:** Association of risk factors vs. types of problems in the combined group with AOR.

		LBP					KP					NP				
Pseudo R^2^, LL		0.1860, −34.410					0.07431, −38.523					0.1456, −33.555				
LL0, LLR p		−42.274, 0.07281					−41.616, 0.7213					−39.273, 0.2469				
	**Characteristics**	**Coef.**	**Std. Error**	**AOR**	**95% CI**	***p***	**Coef.**	**Std. Error**	**AOR**	**95% CI**	***p***	**Coef.**	**Std. Error**	**AOR**	**95% CI**	***p***
Socio-demographic Characteristics	Age	0.1310	0.049	1.14	[0.034, 0.228]	0.008 *	0.0370	0.040	1.03	[−0.041, 0.115]	0.352	0.0229	0.045	1.023	[−0.066, 0.112]	0.613
	Weight	0.0130	0.050	1.01	[−0.085, 0.111]	0.796	0.0249	0.045	1.03	[−0.063, 0.113]	0.577	−0.0815	0.060	0.92	[−0.198, 0.035]	0.171
	BMI	−0.1916	0.163	0.83	[−0.511, 0.128]	0.240	0.0878	0.140	1.09	[−0.187, 0.363]	0.531	0.1554	0.172	1.168	[−0.182, 0.493]	0.366
	Sleep	0.0203	0.184	1.02	[−0.340, 0.381]	0.912	0.1168	0.173	1.12	[−0.222, 0.456]	0.500	−0.6035	0.295	0.546	[−1.181,−0.026]	0.041 *
Behavioural Characteristics	Work experience	−0.0741	0.053	0.93	[−0.179, 0.030]	0.164	−0.0264	0.048	0.98	[−0.120, 0.068]	0.582	0.0594	0.055	1.06	[−0.048, 0.167]	0.279
	Exercise vs. non-exercise	1.1604	0.916	3.2	[−0.635, 2.956]	0.205	−0.0646	0.805	0.94	[−1.642, 1.513]	0.936	0.8174	0.918	2.26	[−0.981, 2.616]	0.373
	Alcoholic vs. non-alcoholic	0.5909	0.761	1.8	[−0.901, 2.083]	0.438	−0.6138	0.709	0.54	[−2.004, 0.777]	0.387	0.2381	0.736	1.26	[−1.204, 1.680]	0.746
	Smokers and tobacco users vs. non-smokers and tobacco users	0.2442	0.807	1.28	[−1.337, 1.826]	0.762	0.0760	0.706	0.93	[−1.308, 1.460]	0.914	−0.8191	0.789	0.44	[−2.366, 0.728]	0.299
Occupation	Regular heavy vehicle driving vs. not	−0.6033	0.878	0.55	[−2.323, 1.117]	0.492	0.0573	0.786	0.95	[−1.483, 1.598]	0.942	−1.3640	0.940	0.255	[−3.207, 0.479]	0.147

Note: CI = Confidence Interval; Pseudo R^2^ = pseudo R-squared; LL = log-likelihood; LL0 = LL-Null; LLR p = LLR *p*-value; * indicates: *p*-value < 0.05 is considered statistically significant.

**Table 5:** Hyperparameter tuning of LR models.

Logistic Regression Models	**Hyperparameter**	**Hyperparameter Space**	**Tuned Value**		
			**LBP**	**KP**	NP *
	Regularization Parameter (C)	0.001, 0.01, 0.1, 1, 10, 100, 1000	0.001	0.01	1.0
	Class Weight Parameter	None, ‘balanced’	None	‘balanced’	None
	Maximum Iterations	100, 200, 300, 400, 500	100	100	100
	Multi Class Classification	‘ovr’, ‘multinomial’	‘multinomial’	‘ovr’	‘auto’
	Penalization	‘l1’, ‘l2’	‘l2’	‘l2’	‘l2’
	Random State	None, 42	42	42	None
	Solver for Optimization	‘newton-cg’, ‘lbfgs’, ‘liblinear’, ‘sag’, ‘saga’	‘sag’	‘newton-cg’	‘lbfgs’
	Warm Start to Speed Up Convergence	False, True	False	False	False

* Default hyperparameter values.

**Table 6:** Evaluation metrics.

		LBP	KP	NP
Training	Accuracy	0.69	0.63	0.68
	Precision	0.69	0.55	0.54
	Recall	0.71	0.66	0.77
	F1 Score	0.69	0.59	0.63
	ROC AUC	0.72	0.68	0.74
	Cost Function	0.62	0.65	0.59
	Cross-Validation Accuracy	0.687	0.559	0.657
Test	Accuracy	0.63	0.56	0.59
	Precision	0.73	0.47	0.4
	Recall	0.54	0.64	0.45
	F1 Score	0.62	0.54	0.42
	ROC AUC	0.65	0.65	0.47
	Cost Function	0.65	0.68	0.87

## Data Availability

Data are available to readers upon request.
